# Misrepresentations and Flawed Logic About the Prevalence of False Memories

**DOI:** 10.1002/acp.3265

**Published:** 2016-10-14

**Authors:** Robert A. Nash, Kimberley A. Wade, Maryanne Garry, Elizabeth F. Loftus, James Ost

**Affiliations:** ^1^ Aston UniversityBirminghamUK; ^2^ University of WarwickCoventryUK; ^3^ Victoria University of WellingtonWellingtonNew Zealand; ^4^ University of CaliforniaIrvineUSA; ^5^ University of PortsmouthPortsmouthUK

## Abstract

Brewin and Andrews (2016) propose that just 15% of people, or even fewer, are susceptible to false childhood memories. If this figure were true, then false memories would still be a serious problem. But the figure is higher than 15%. False memories occur even after a few short and low‐pressure interviews, and with each successive interview, they become richer, more compelling, and more likely to occur. It is therefore dangerously misleading to claim that the scientific data provide an “upper bound” on susceptibility to memory errors. We also raise concerns about the peer review process. © 2016 The Authors Applied Cognitive Psychology Published by John Wiley & Sons Ltd.

Decades of research shows that people can come to remember events that never happened. In fact, as scientists who conduct this kind of research, we know it is not all that difficult to ‘implant’ false memories. Many participants in our experiments remember a wide range of experiences that just were not so. It is puzzling, therefore, that Brewin and Andrews (2016) say it is hard to implant false memories, that false memories are rare, and that false memory rates are overstated.

If Brewin and Andrews (2016) want to make broad, sweeping conclusions about this literature, then they should have obtained the original data from as many researchers as possible, combined those data, and recoded them. They could have done exactly that—after all, they contacted several researchers for information in the course of writing their paper.

But thankfully, others have already undertaken this task. Scoboria et al. (submitted) recently conducted a mega‐analysis in which they combined and recoded the data from eight published memory implantation studies. They found that, overall, 22% of subjects reported either ‘complete’ or ‘substantial’ false memories, and a further 9% reported ‘partial’ false memories. Put another way, nearly a third of people came to remember something about an event that never happened. The fact that more than a fifth of people in Scoboria et al.'s analysis, or 15% in Brewin and Andrews' analysis, developed rich false memories packed with perceptual detail is somewhat astounding given that some of these memories were elicited under experimental conditions designed to produce low false‐memory rates (e.g., Ost, Foster, Costall, & Bull, 2005, and the control conditions in studies such as Desjardins & Scoboria, 2007, and Hyman & Pentland, 1996).

Brewin and Andrews (2016) also complain that almost no one in an imagination inflation study experiences false memories. But the aim of imagination inflation research is not usually to create false memories. Instead, it is to investigate the ways in which imagining counterfactual childhood events can inflate people's confidence that those events were real. In fact, Brewin and Andrews themselves admit that this work is “by and large only intended to assess autobiographical belief” (p.17).

Now, perhaps at this point, a critic might charge that the literature we have just presented confirms that most people are *not* susceptible to the influences that create false memories or false beliefs. That is essentially what Brewin and Andrews (2016) themselves have said. But such a claim fundamentally misrepresents scientific understanding. After all, let us consider the differences between laboratory paradigms and the real‐world situations they parallel. When we conduct these memory implantation studies, we do not pair an authoritative therapist with a vulnerable client. We are not spending weeks and weeks suggesting to a vulnerable client—who is searching for reasons for her current problems—that she has buried childhood trauma in some dark corner of her memory. Instead, we pair a 20‐something‐year‐old graduate student with a volunteer. They meet a few times over the course of a week or two, while the graduate student encourages the volunteer to try and remember something that did not happen. The fact that such a weak analog of dubious recovered‐memory techniques produces false memories *at any rate*, let alone in a sizable minority, is astonishing.

Moreover, study after study shows that what begins merely as a subject's willingness to entertain a suggestion often develops over the course of the study into a richer and compelling recollection. People's ratings of the characteristics of their own memories also increase in parallel with independent judges' ratings. We can ponder for a moment what would happen as the number of sessions increased to approximate the number of times someone would see a therapist. The smart money is on the prediction that the rate of false memories and the strength of self‐report ratings would increase.

It is clear that there are copious reasons to predict that the rates of false memory reported in these studies do not represent an upper bound on people's susceptibility to suggestion. But let us stop to consider the counterfactual for a moment. What if Brewin and Andrews were right? What would it mean if only 15% of people, or even fewer, were susceptible to wholly false memories? Brewin and Andrews (2016) repeatedly imply that because false memories (apparently) occur in only a tiny fraction of people, scientists are making a mountain out of a molehill. But let us look at this finding another way. If there were a drug on the market that produced ‘adverse events’ at even the low, incorrect rate the authors suggest, it would be taken off the market in a flash.

But let us stay in the realm of psychology. Suppose a clinical disorder occurred in 15% of the general population. We would consider that disorder to be highly prevalent. Would Brewin and Andrews? Given their longstanding interest in trauma, let us consider a concrete example, such as Posttraumatic Stress Disorder (PTSD). The lifetime prevalence of PTSD among adult Americans is estimated at 6.8%. That is less than half of the incorrect rate of false memories that Brewin and Andrews (2016) estimate—but they quite rightfully do not relegate PTSD to molehill status. How about other serious psychological disorders? Many serious psychological disorders have a prevalence of under 3%, and some even less than 1%. But if 1 person in 100 has a disorder that seriously affects the quality of their life and ability to function, that is a huge number in absolute terms at the level of the population. So even if 15% were a correct figure, it is still hard to understand how Brewin and Andrews could marshal an argument that 15% is not worth worrying about.

Brewin and Andrews (2016) also take to task scholars who—in their writings and expert testimony—talk about the fallibility of memory. But these scholars know a lot more about memory fallibility than just the three paradigms that Brewin and Andrews dissect. For instance, memory scholars know about self‐enhancing memory distortions, such as Bahrick, Hall, and Berger's (1996) famous work showing that people remember their school grades as better than they actually were. They know about studies of people who came to remember they were abused, but later realized their memories were false (Lief & Fetkewicz, 1995; De Rivera, 2000). These scholars know about research with subjects who remember being abducted by aliens, for whom these memories feel entirely real (McNally et al., 2004). These scholars also know the many studies of misinformation showing that people's memories are easily and quickly distorted (see Schacter & Loftus, 2013, for a review). In short, scholarly opinions about the fragility of memory derive from many different types of studies that go far beyond what Brewin and Andrews reviewed.

Considered as a whole, Brewin and Andrews' (2016) paper ignores the facts so blithely that it seems more suited to being called an Op‐Ed piece than a peer reviewed ‘Research Article’. Maybe that is because it probably was not peer reviewed. We asked both the founding editor and the editor who invited our commentary whether Brewin and Andrews' paper had been sent out for peer review. They did not answer. We also contacted 21 false‐memory experts whom Brewin and Andrews cited, and we asked these experts if they would be willing to confirm that—just like the five of us—they did *not* review the original submission for ACP. Each of them answered: none of them had reviewed it. These data fit with other data on the interval between when Brewin and Andrews first submitted this paper and when it was accepted. A close look at the publication history reveals a curiously short interval: just 28 days, which apparently included sufficient time for revisions and resubmission. But perhaps such a short turnaround time means that *Applied Cognitive Psychology* has recently committed itself to extremely speedy peer review. To address this possibility, we examined all papers appearing in the first three 2016 issues of ACP that were designated as a ‘Research Article’ (like Brewin and Andrews' paper). Among these 39 papers, the mean turnaround was 282 days, a figure more than 10 times the 28 days Brewin and Andrews enjoyed. The median turnaround was 261 days, with a range of 101–699 days. In other words, even the paper with the next quickest turnaround still took nearly four times as long.

Of course, it goes without saying that peer review is crucial. Because of that fact, scholarly publishers worldwide have embraced the Code of Conduct put forth by the worldwide Committee on Publication Ethics (COPE). More than 900 journals and dozens of publishers are members, who choose to comply with the COPE Code, and its Principles of Transparency and Best Practice in Scholarly Publishing. The very first Principle says:

Peer review process: Journal content must be clearly marked as whether peer reviewed or not. Peer review is defined as obtaining advice on individual manuscripts from reviewers expert in the field who are not part of the journal's editorial staff. This process, as well as any policies related to the journal's peer review procedures, shall be clearly described on the journal's Web site (Committee on Publication Ethics, 2014)

Some of our science's best publishing houses and journals refer to COPE. For example, Elsevier obliged all its journals (including the Journal of Applied Research in Memory & Cognition) to follow COPE principles. Wiley‐Blackwell has, for some reason, left this decision to each journal. As a result of that decision, what are we commenting on now? A flawed opinion piece masquerading as a peer reviewed article.

## References

[acp3265-bib-0001] Bahrick, H. P. , Hall, L. K. , & Berger, S. A. (1996). Accuracy and distortion in memory for high school grades. Psychological Science, 7, 265–271.

[acp3265-bib-0002] Brewin, C. R. , & Andrews, B. (2016). Creating memories for false autobiographical events in childhood: A systematic review. Applied Cognitive Psychology. doi: 10.1002/acp.3220 10.1002/acp.3220 PMC5248593 28163368

[acp3265-bib-0003] Committee on Publication Ethics (2014). Principles of transparency and best practice in scholarly publishing. Retrieved from http://publicationethics.org/files/Principles_of_Transparency_and_Best_Practice_in_Scholarly_Publishingv2.pdf. Accessed 23rd May 2016

[acp3265-bib-0004] De Rivera, J. (2000). Understanding persons who repudiate memories recovered in therapy. Professional Psychology: Research and Practice, 31, 378–386.

[acp3265-bib-0005] Desjardins, T. , & Scoboria, A. (2007). “You and your best friend Suzy put Slime in Ms. Smollett's desk”: Producing false memories with self‐relevant details. Psychonomic Bulletin & Review, 14, 1090–1095. 18229480 10.3758/bf03193096

[acp3265-bib-0006] Lief, H. I. , & Fetkewicz, J. (1995). Retractors of false memories: The evolution of pseudo‐memories. Journal of Psychiatry & Law, 23, 411–435.

[acp3265-bib-0007] Hyman, I. E. Jr. , & Pentland, J. (1996). The role of mental imagery in the creation of false childhood memories. Journal of Memory and Language, 35, 101–117.

[acp3265-bib-0008] McNally, R. J. , Lasko, N. B. , Clancy, S. A. , Macklin, M. L. , Pitman, R. K. , & Orr, S. P. (2004). Psychophysiological responding during script‐driven imagery in people reporting abduction by space aliens. Psychological Science, 15, 493–497. 15200635 10.1111/j.0956-7976.2004.00707.x

[acp3265-bib-0009] Ost, J. , Foster, S. , Costall, A. , & Bull, R. (2005). False reports of childhood events in appropriate interviews. Memory, 13, 700–710. 16191820 10.1080/09658210444000340

[acp3265-bib-0010] Schacter, D. L. , & Loftus, E. F. (2013). Memory and law: What can cognitive neuroscience contribute? Nature Neuroscience, 16, 119–123. 23354384 10.1038/nn.3294

[acp3265-bib-0011] Scoboria, A. , Wade, K. A. , Lindsay, D. S. , Azad, T. , Strange, D. , Ost, J. , & Hyman, I. . (submitted). Recoding and reanalyzing memory reports from eight previously published false memory studies. Manuscript submitted for publication.

